# Supplementary material for: Global Landscape Review of Serotype-Specific Invasive Pneumococcal Disease Surveillance among Countries Using PCV10/13: The Pneumococcal Serotype Replacement and Distribution Estimation (PSERENADE) Project
Source: Microorganisms. 2021 Apr 2;9(4):742. doi: 10.3390/microorganisms9040742 (PMC8066045; doi:10.3390/microorganisms9040742)
Supplement: Supplementary file 1 [file microorganisms-09-00742-s001.pdf]

Global landscape review of serotype-specific invasive pneumococcal disease surveillance among countries using PCV10/13: The Pneumococcal Serotype Replacement and Distribution Estimation (PSERENADE) Project

Table of Contents

Search Terms .... **Error! Bookmark not defined.**

Table S1. Surveillance data sources for sites in PSERENADE. 5

Table S2. PCV-using countries with IPD surveillance, whether included in PSERENADE and if so whether incidence data were provided 9

Table S3. Characteristics of data in PSERENADE by surveillance site. 12

References .... **Error! Bookmark not defined.**7

Search Terms

EMBASE (with Medline)

1. Streptococcus pneumoniae/
2. pneumococcal infection/
3. pneumococcal meningitis/
4. Streptococcus pneumonia/
5. Pneumococcus vaccine/
6. streptococcus pneumoniae.mp.
7. pneumococcus.mp.
8. s pneumoniae.mp.
9. pneumococci.mp.
10. streptococcus pneumoniae meningitis.mp.
11. pneumococcal meningitis.mp.
12. pneumococcal bacter?emia.mp.
13. pneumococcal pneumonia.mp.
14. invasive pneumococcal.mp.
15. pneumococcal conjugate.mp.
16. pneumococc* vaccin*.mp.
17. pcv?10.mp.
18. pcv?13.mp.
19. prev?nar.mp.
20. synflorix.mp.
21. ten?valent.mp.
22. thirteen?valent.mp.
23. 10?valent.mp.
24. 13?valent.mp.
25. 1 OR 2 OR 3 OR 4 OR 5 OR 6 OR 7 OR 8 OR 9 OR 10 OR 11 OR 12 OR 13 OR 14 OR 15 OR 16 OR 17 OR 18 OR 19 OR 20 OR 21 OR 22 OR 23 OR 24
26. serotype/
27. sero?type*.mp.
28. serogroup*.mp.
29. vaccine?type.mp.
30. 26 OR 27 OR 28 OR 29
31. 25 AND 30
32. Limit 40 to "human and yr=2011–Current"

PubMed

((Streptococcus pneumoniae [MeSH] OR Pneumococcal Infections [MeSH] OR Bacteremia [MeSH] OR Pneumococcal Vaccines [MeSH] OR "Streptococcus pneumoniae"[tw] OR pneumococcus[tw] OR "s pneumoniae"[tw] OR pneumococci[tw] OR "pneumococcal meningitis"[tw] OR "pneumococcal bacteremia"[tw] OR "pneumococcal bacteraemia"[tw] OR "pneumococcal pneumonia"[tw] OR "invasive pneumococcal"[tw] OR "pneumococcal conjugate"[tw] OR pneumococc*[tw] vaccine*[tw] OR pcv10[tw] OR "pcv 10"[tw] OR "pcv-10"[tw] OR pcv13[tw] OR "pcv 13"[tw] OR "pcv-13"[tw] OR prevnar[tw] OR prevenar[tw] OR synflorix[tw] OR "ten valent"[tw] OR "ten-valent"[tw] OR "thirteen valent"[tw] OR "thirteen-valent"[tw] OR "10valent"[tw] OR "10 valent"[tw] OR "10-valent"[tw] OR "13valent"[tw] OR "13 valent"[tw] OR "13-valent"[tw]) AND (Serogroup [MeSH] OR serotype*[tw] OR sero type*[tw] OR

serogroup*[tw] OR vaccine type*[tw] OR vaccine-type*[tw])) AND (("2011/01/01"[PDAT] : "2018/12/31"[PDAT]) AND "humans"[MeSH Terms])

Global Health Database

1. Streptococcus pneumoniae.mp. OR pneumococcus.mp. OR s pneumoniae.mp. OR pneumococci.mp. OR pneumococcal conjugate.mp. OR pneumococc* vaccin*.mp. OR streptococcus pneumoniae meningitis.mp. OR pneumococcal meningitis.mp. OR pneumococcal bacter?emia.mp. OR pneumococcal pneumonia.mp. OR invasive pneumococcal.mp. OR pcv?10.mp. OR pcv?13.mp. OR prev?nar.mp. OR synflorix.mp. OR ten?valent.mp. OR thirteen?valent.mp. OR 10?valent.mp. OR 13?valent.mp.
2. sero?type*.mp. OR serogroup*.mp. OR vaccine?type.mp.
3. 1 AND 2
4. Limit 3 to yr="2011–Current"

Web of Science (All Databases)

1. ((Streptococcus NEAR/0 pneumoniae) OR pneumococcus OR (s NEAR/0 pneumoniae) OR pneumococci OR (pneumococcal NEAR/0 conjugate) OR (pneumococc* NEAR/0 vaccin*) OR (streptococcus NEAR/0 pneumoniae NEAR/1 meningitis) OR (pneumococcal NEAR/0 meningitis) OR (pneumococcal NEAR/0 bacteremia) OR (pneumococcal NEAR/0 bactaeremia) OR (pneumococcal NEAR/0 pneumonia) OR (invasive NEAR/0 pneumococcal) OR pcv10 OR pcv 10 OR pcv-10 OR pcv13 OR pcv 13 OR pcv-13 OR prevenar OR prevnar OR synflorix OR ten valent OR ten-valent OR thirteen valent OR thirteen-valent OR 10valent OR 10 valent OR 10-valent OR 13valent OR 13 valent OR 13-valent)
2. serotype* OR (sero NEAR/0 type*) OR serogroup* OR vaccine type OR vaccine type OR vaccine-type
3. 1 AND 2
4. Limit 3 to yr="2011–2018"

Global Index Medicus

1. ((Streptococcus pneumoniae OR pneumococcus OR s pneumoniae OR pneumococci OR pneumococcal conjugate OR pneumococc* vaccin* OR streptococcus pneumoniae meningitis OR pneumococcal meningitis OR pneumococcal bacteremia OR pneumococcal bacteraemia OR pneumococcal pneumonia OR invasive pneumococcal OR pcv10 OR pcv 10 OR pcv-10 OR pcv13 OR pcv 13 OR pcv-13 OR prevenar OR prevnar OR synflorix OR ten valent OR ten-valent OR thirteen valent OR thirteen-valent OR 10valent OR 10 valent OR 10-valent OR 13valent OR 13 valent OR 13-valent) AND (serotype* OR sero type* OR serogroup* OR vaccine type OR vaccine type OR vaccine-type))
2. Year limits: 2011–2018

Pascal

1. ("Streptococcus pneumoniae" OR pneumococcus OR "s pneumoniae" OR pneumococci OR "pneumococcal conjugate" OR "pneumococc* vaccin*" OR "streptococcus pneumoniae meningitis" OR "pneumococcal meningitis" OR "pneumococcal bacter*emia" OR "pneumococcal pneumonia" OR "invasive pneumococcal" OR pcv*10 OR pcv*13 OR prev*nar OR synflorix OR ten*valent OR thirteen*valent OR 10*valent OR 13*valent)

2. (sero*type* OR serogroup* OR vaccine*type)
3. 1 AND 2
4. 2011 TO 2018

Africa Wide Information

1. ((Streptococcus pneumoniae OR pneumococcus OR s pneumoniae OR pneumococci OR pneumococcal conjugate OR pneumococc* vaccin* OR streptococcus pneumoniae meningitis OR pneumococcal meningitis OR pneumococcal bacteremia OR pneumococcal bacteraemia OR pneumococcal pneumonia OR invasive pneumococcal OR pcv10 OR pcv 10 OR pcv-10 OR pcv13 OR pcv 13 OR pcv-13 OR prevenar OR prevnar OR synflorix OR ten valent OR ten-valent OR thirteen valent OR thirteen-valent OR 10valent OR 10 valent OR 10-valent OR 13valent OR 13 valent OR 13-valent) AND (serotype* OR sero type* OR serogroup* OR vaccine type OR vaccine type OR vaccine-type))
2. Year limit = 2011–2018

Table S1. Surveillance data sources for sites in PSERENADE

Region	Site	Name of Surveillance Site or Network
North America	Canada, Alberta	Calgary Area Streptococcus pneumoniae Epidemiology Research (CASPER)
	Canada, Ontario	Toronto Invasive Bacterial Diseases Network (TIBDN)
	Canada, Quebec (excluding Nunavik)	Quebec National Public Health Institute (Institut national de santé publique du Québec)
	Canada, Quebec-Nunavik	
	USA, ABCs	Active Bacterial Core Surveillance (ABCs)
	USA, Alaska	Arctic Investigations Program (AIP)
	USA, California	Kaiser Permanente Northern California (KPNC)
	USA, Massachusetts	Massachusetts Department of Public Health (MDPH)
	USA, Southwest (Indigenous)	The Johns Hopkins Center for American Indian Health Active Bacterial Surveillance System
	USA, Utah	University of Utah and Intermountain Healthcare
Latin America and the Caribbean	Argentina	Servicio Bacteriología Clínica, Departamento de Bacteriología, INEI -ANLIS "Dr. Carlos G. Malbrán" - Laboratorio Nacional de Referencia de *S. pneumoniae*.
	Bolivia (SIREVA)	SIREVA II Network[1]
	Brazil	National Reference Laboratory for Meningitis and Pneumococcal Infections, Institute Adolfo Lutz, Center of Bacteriology, São Paulo, State of São Paulo, Brazil
	Chile, Metropolitan Region	Instituto de Salud Pública de Chile
	Chile, Non-Metropolitan Regions	
	Colombia (SIREVA)	Colombia SIREVA II[2]
	Costa Rica	Centro Nacional de Referencia de Bacteriología
	Dominican Republic (SIREVA)	SIREVA II Network[1]
	Ecuador (SIREVA, WHO)	SIREVA II Network[1] and WHO Global IB-VPD Surveillance Network[3]
	El Salvador (SIREVA, WHO)	SIREVA II Network[1] and WHO Global IB-VPD Surveillance Network[3]
	Guatemala (SIREVA)	SIREVA II Network[1]
	Honduras (SIREVA, WHO)	SIREVA II Network[1] and WHO Global IB-VPD Surveillance Network[3]
	Mexico (SIREVA)	SIREVA II Network[1]
	Nicaragua (SIREVA, WHO)	SIREVA II Network[1] and WHO Global IB-VPD Surveillance Network[3]
	Panama (SIREVA)	SIREVA II Network[1]
	Paraguay	Laboratorio Central de Salud Pública
	Peru (SIREVA, WHO)	SIREVA II Network[1] and WHO Global IB-VPD Surveillance Network[3]
	Uruguay (SIREVA)	SIREVA II Network[1]
	Venezuela (SIREVA)	SIREVA II Network[1]
Europe	Austria (ECDC)	ECDC[4]
	Belgium	Belgian National Reference Center of Streptococcus Pneumoniae
	Bulgaria	Medical University of Sofia, Bulgaria
	Czech Republic	National Reference Laboratory for Streptococcal Infections (NIPH)
	Denmark	IPD Surveillance, Statens Serum Institut (SSI)
	Finland	National Institute for Health and Welfare (THL)
	France	Epibac Santé Publique France and Pneumococcus Regional Observatory - National Reference Laboratory-Pneumococcus Regional Observatory (ORP-CNRP)
	Germany	German National Reference Center for Streptococci (GNRCS)
	Greece	Department for Epidemiological Surveillance and Intervention of the National Public Health Organization and National Reference Laboratory for Meningitis
	Iceland	Landspitali University Hospital
	Ireland	Irish Meningitis and Sepsis Reference Laboratory (IMSRL); Health Protection Surveillance Centre (HPSC)

	Italy	Italian National Institute of Health (Istituto Superiore di Sanità, ISS
	Latvia	The Centre for Disease Prevention and Control of Latvia (CDPC)
	Lithuania (ECDC)	ECDC ECDC[4]
	Netherlands	Netherlands Reference Laboratory for Bacterial Meningitis (NRLBM)
	Norway	Norwegian Surveillance System for Communicable Diseases
	Poland	National Reference Centre for Bacterial Meningitis
	Slovakia	Slovak National SurveillanceReference Center for Pneumococcal and Haemophilus Diseases
	Slovenia	National Institute of Public Health (Nacionalni inštitut za javno zdravje)
	Spain, Catalonia	Public Health Agency of Catalonia
	Spain, Madrid	Consejería de Sanidad de la Comunidad de Madrid
	Spain, Navarra	Instituto de Salud Pública de Navarra
	Sweden	The Public Health Agency of Sweden
	Switzerland	Swiss Federal Office of Public Health (FOPH)
	UK, England	Immunisation and Countermeasures Division and Respiratory and Vaccine Preventable Bacteria Reference Unit (RVPBRU), Public Health England
	UK, Scotland	Health Protection Scotland Pneumococcal Invasive Disease Enhanced Reporting (SPIDER)
**Sub-Saharan Africa**	Benin (WHO)	WHO Global IB-VPD Surveillance Network[3]
	Cameroon (WHO)	WHO Global IB-VPD Surveillance Network[3]
	Ethiopia (WHO)	WHO Global IB-VPD Surveillance Network[3]
	Kenya, Asembo	Kenya Medical Research Institute (KEMRI) and US CDC Population Based Infectious Disease Surveillance (PBIDS)
	Kenya, Kibera	
	Kenya, Kilifi	KEMRI-Wellcome Trust Research Programme (KWTRP)
	Madagascar (WHO)	WHO Global IB-VPD Surveillance Network[3]
	Malawi, Blantyre District	Malawi-Liverpool-Wellcome Trust Clinical Research Programme (MLW)
	South Africa	Group for Enteric, Respiratory, and Meningeal Disease Surveillance in South Africa (GERMS-SA)
	The Gambia, Basse	Basse Health and Demographic Surveillance System (BHDSS)
	Zimbabwe (WHO)	WHO Global IB-VPD Surveillance Network[3]
**Northern Africa & Western Asia**	Israel	Israeli Pediatric Bacteremia and Meningitis Group
	Morocco, Grand Casablanca	Ibn Rochd University Hospital Centre of Casablanca
**Asia**	Bangladesh	Dhaka Shishu Hospital (DSH), Shishu Shasthya Foundation Hospital, & Kumudini Women's Medical College Hospital
	Hong Kong	Public Health Laboratory Services Branch Centre (PHLSBC) Hong Kong
	Japan	Pediatric IPD Surveillance Group, Japan and Adult IPD Surveillance Group, Japan
	Mongolia	National Center of Communicable Diseases (NCCD), Ministry of Health, Mongolia
	Singapore	Singapore Ministry of Health
**Oceania**	Australia (Non-Indigenous)	National Notifiable Diseases Surveillance System (NNDSS) of the Communicable Diseases Network Australia (CDNA)
	Australia, Northern Territory	
	Fiji	Fiji IB-VPD New Vaccine Evaluation Project
	New Zealand	Institute of Environmental Science and Research (ESR)

[1] Sistema de Redes de Vigilancia de los Agentes responsables de Neumonias y Meningitis (SIREVA II) Network Reports (2006-2016) [1]. [2] Vigilancia por Laboratorio de aislamientos invasores de Streptococcus pneumoniae Colombia 2006-2018 (SIREVA II) [2]. [3] WHO Global Invasive Bacterial Vaccine Preventable Disease (IB-VPD) Surveillance Network; Some sites had data from both the SIREVA and WHO Global IB-VPD networks to compile the most up-to-date data for all age groups [1,3]. [4] European Centre for Disease Prevention and Control (ECDC) European Surveillance System [4]

Table S2. PCV-using countries with IPD surveillance, whether included in PSERENADE and if so whether incidence data were provided. Y = yes; N = no.

Region	Country	Eligible for PSERENADE	Included in PSERENADE	Has incidence data (among PSERENADE sites)
North America	Canada	Y	Y	Y
	United States	Y	Y	Y
Latin America and the Caribbean	Argentina	Y	Y	N
	Bolivia	Y	Y	N
	Brazil	Y	Y	Y
	Chile	Y	Y	Y
	Colombia	Y	Y	N
	Costa Rica	Y	Y	N
	Dominican Republic	Y	Y	N
	Ecuador	Y	Y	N
	El Salvador	Y	Y	N
	Guatemala	Y	Y	N
	Honduras	Y	Y	N
	Mexico	Y	Y	N
	Nicaragua	Y	Y	N
	Panama	Y	Y	N
	Paraguay	Y	Y	N
	Peru	Y	Y	N
	Trinidad and Tobago	Y	N	--
	Uruguay	Y	Y	N
	Venezuela	Y	Y	N
Europe	Austria	Y	Y	N
	Belgium	Y	Y	Y
	Bulgaria	Y	Y	N
	Czech Republic	Y	Y	Y
	Denmark	Y	Y	Y
	Finland	Y	Y	Y
	France	Y	Y	Y
	Germany	Y	Y	Y
	Greece	Y	Y	Y
	Hungary	N	--	--
	Iceland	Y	Y	Y
	Ireland	Y	Y	Y
	Italy	Y	Y	Y
	Latvia	Y	Y	Y
	Lithuania	Y	Y	N
	Luxembourg	N	--	--
	Netherlands	Y	Y	Y
	Norway	Y	Y	Y
	Poland	Y	Y	Y
	Portugal	Y	N	--
	Slovakia	Y	Y	Y
	Slovenia	Y	Y	Y
	Spain	Y	Y	Y
	Sweden	Y	Y	Y
	Switzerland	Y	Y	Y

	United Kingdom	Y	Y	Y
**Sub-Saharan Africa**	Angola	N	--	--
	Benin	Y	Y	N
	Burkina Faso	Y	N	--
	Burundi	N	--	--
	Cote d'Ivoire	N	--	--
	Cameroon	Y	Y	N
	Central African Republic	N	--	--
	Democratic Republic of the Congo	N	--	--
	Eritrea	N	--	--
	eSwatini	N	--	--
	Ethiopia	Y	Y	N
	Gambia, The	Y	Y	Y
	Ghana	Y	N	--
	Kenya	Y	Y	Y
	Lesotho	N	--	--
	Madagascar	Y	Y	N
	Malawi	Y	Y	Y
	Mali	Y	N	--
	Mozambique	Y	N	--
	Namibia	N	--	--
	Niger	N	--	--
	Nigeria	N	--	--
	Rwanda	N	--	--
	Senegal	N	--	--
	Sierra Leone	N	--	--
	South Africa	Y	Y	Y
	Tanzania	N	--	--
	Togo	Y	N	--
	Uganda	N	--	--
	Zambia	N	--	--
	Zimbabwe	Y	Y	N
**Northern Africa & Western Asia**	Armenia	N	--	--
	Azerbaijan	N	--	--
	Bahrain	N	--	--
	Cyprus	Y	N	--
	Georgia	N	--	--
	Israel	Y	Y	Y
	Kuwait	Y	N	--
	Morocco	Y	Y	Y
	Oman	N	--	--
	Qatar	N	--	--
	Saudi Arabia	Y	N	--
	Sudan	N	--	--
	Turkey	Y	N	--
	Yemen	Y	N	--
**Asia**	Afghanistan	N	--	--
	Bangladesh	Y	Y	Y
	Cambodia	N	--	--
	Hong Kong	Y	Y	Y
	Japan	Y	Y	Y

	Mongolia	Y	Y	Y
	Nepal	N	--	--
	Pakistan	N	--	--
	Philippines	N	--	--
	Republic of Korea	Y	N	--
	Singapore	Y	Y	Y
	Taiwan	Y	N	--
	Uzbekistan	N	--	--
**Oceania**	Australia	Y	Y	Y
	Fiji	Y	Y	Y
	New Zealand	Y	Y	Y
	Papua New Guinea	N	--	--

Table S3. Characteristics of data in PSERENADE by surveillance site.

Region[1]	Site[2]	Availability of data[3]							Specimen collected[4]	Additional pneumococcal detection methods[5]	Serotyping methods[6]
		0–17 years	≥18 years	Pre-PCV	PCV7 period	PCV10/13 period	Incidence data	Clinical syndrome linked			
N. Am.	Canada, Alberta	Y	Y	Y	Y	Y	Y	Y	B, CSF, PF	--	Q
	Canada, Ontario	Y	Y	Y	Y	Y	Y	Y	B, CSF, PF	--	Q
	Canada, Quebec	Y	Y	Y	Y	Y	Y	N	B, CSF, PF	Nuc	Q
	USA, ABCs	Y	Y	Y	Y	Y	Y	Y	B, CSF, PF	--	Q, PCR70, WGS
	USA, Alaska	Y	Y	Y	Y	Y	Y	Y	B, CSF, PF	--	Q, L, PCR70
	USA, California	Y	Y	Y	Y	Y	Y	Y	B, CSF, PF	--	Q
	USA, Massachusetts	Y	N	N	Y[7]	Y[7]	Y	Y	B, CSF	--	Q
	USA, Southwest (Indigenous)	Y	Y	Y	Y	Y	Y	Y	B, CSF	--	Q
	USA, Utah	Y	N	N	N	Y[7]	Y	Y	B, CSF, PF	Nuc	Q
LA & C	Argentina	Y	Y	Y	--	Y	N	Y	B, CSF, PF	Nuc	Q, PCR37, PCR70
	Bolivia (SIREVA)	Y	Y	Y	--	Y	N	N[8]	B, CSF, PF	Ag	Q
	Brazil	Y	Y	Y	--	Y	Y[9]	Y	B, CSF, PF	Nuc	Q, L, PCR70
	Chile, Metropolitan Region	Y	Y	Y	Y	Y	Y	Y	B, CSF, PF	Nuc	Q
	Chile, Non-Metropolitan Regions	Y	Y	Y	--	Y	Y	Y	B, CSF, PF	Nuc	Q
	Colombia (SIREVA)	Y	Y	Y	--	Y	N	N[8]	B, CSF, PF	Nuc, Ag	Q, PCR37
	Costa Rica	Y	Y	Y	Y	Y	N	Y	B, CSF, PF	Nuc	Q
	Dominican Republic (SIREVA)	Y	Y	Y	--	Y	N	N[8]	B, CSF, PF	Nuc, Ag	Q, PCR37
	Ecuador (SIREVA, WHO)	Y	Y	Y	Y	Y	N	Y	B, CSF	Nuc, Ag	Q, PCR37
	El Salvador (SIREVA, WHO)	Y	Y	Y	Y	Y	N	Y	B, CSF, PF	Ag	Q
	Guatemala (SIREVA)	Y	Y	Y	--	Y	N	N[8]	B, CSF, PF	Nuc, Ag	Q
	Honduras (SIREVA, WHO)	Y	Y	Y	--	Y	N	Y	B, CSF, PF	Ag	Q
	Mexico (SIREVA)	Y	Y	Y	Y	Y	N	N[8]	B, CSF, PF	Nuc, Ag	Q, PCR37, PCR70
	Nicaragua (SIREVA, WHO)	Y	Y	Y	--	Y	N	Y	B, CSF, PF	Nuc, Ag	Q, PCR37
	Panama (SIREVA)	Y	Y	Y	Y	Y	N	N[8]	B, CSF, PF	Nuc, Ag	Q, PCR37
	Paraguay	Y	Y	Y	--	Y	N	Y	B, CSF, PF	Nuc	Q, L, PCR37, PCR70

	Peru (SIREVA, WHO)	Y	Y	Y	Y	Y	N	Y	B, CSF, PF	Nuc, Ag	Q, PCR37
	Uruguay (SIREVA)	Y	Y	Y	Y	Y	N	N[8]	B, CSF, PF	Nuc, Ag	Q, PCR37
	Venezuela (SIREVA)	Y	Y	Y	--	Y	N	N[8]	B, CSF, PF	Nuc, Ag	Q, PCR70
**Europe**	Austria (ECDC)	Y	Y	N	N	Y	N	Y	B, CSF	Nuc	Q
	Belgium	Y	Y[10]	N	Y	Y	Y	Y	B, CSF, PF	--	Q
	Bulgaria	Y	Y	N	--	Y	N	Y	B, CSF, PF	--	Q, L, PCR37
	Czech Republic	Y	Y	Y	--	Y	Y	Y	B, CSF, PF	Nuc	Q, PCR70
	Denmark	Y	Y	Y	Y	Y	Y	Y	B, CSF, PF	Nuc, Ag	Q, L, PCR70
	Finland	Y	Y	Y	--	Y	Y	Y	B, CSF	Nuc, Ag	Q, L, PCR, WGS
	France	Y	Y	Y	Y	Y	Y	N	B, CSF	--	Q, L, PCR70
	Germany	Y	Y	Y[7]	Y[7]	Y	Y	Y	B, CSF, PF	Nuc, Ag	Q, PCR70
	Greece	Y	Y	N	--	Y	Y[9]	Y	B, CSF	Nuc	Q, PCR17, CST
	Iceland	Y	Y	Y	--	Y	Y	N	B, CSF	Nuc, Ag	L, PCR
	Ireland	Y	Y	Y	Y	Y	Y	Y	B, CSF	Nuc, Ag	Q, L, PCR
	Italy	Y	Y	N	N	Y	Y	Y	B, CSF, PF	Nuc, Ag	Q, L
	Latvia	Y	Y	N	Y[7]	Y	Y	Y	B, CSF, PF	Nuc	Q, L, CST
	Lithuania (ECDC)	Y	Y	N	N	Y	N	Y	Unknown[11]	Nuc, Ag[11]	Unknown[11]
	Netherlands	Y	Y	Y	Y	Y	Y	Y	B, CSF	Nuc	Q, L
	Norway	Y	Y	Y	Y	Y	Y	N	B, CSF, PF	Nuc, Ag	Q, NGS
	Poland	Y	Y	Y	--	Y	Y	Y	B, CSF, PF	Nuc	Q, L, PCR70
	Slovakia	Y	Y	Y	Y	Y	Y	Y	B, CSF, PF	Nuc, Ag	Q, L, PCR70
	Slovenia	Y	Y	Y	--	Y	Y	Y	B, CSF	Nuc	Q, CST
	Spain, Catalonia	Y	Y	N	Y	Y	Y	Y	B, CSF, PF	Nuc, Ag	Q, PCR37
	Spain, Madrid	Y	Y	N	Y	Y	Y	Y	B, CSF, PF	Nuc	Q, L, PCR76
	Spain, Navarra	Y	Y	Y	Y	Y	Y	Y	B, CSF, PF	Nuc, Ag	Q, PCR76
	Sweden	Y	Y	Y	Y	Y	Y	N	B, CSF, PF	Nuc, Ag	Q, L, GD
	Switzerland	Y	Y	Y	Y	Y	Y	Y	B, CSF, PF	Nuc, Ag	Q
	UK, England	Y	Y	Y	Y	Y	Y	N	B, CSF, PF	Nuc	Q, L

	UK, Scotland	Y	Y	Y	Y	Y	Y	N	B, CSF, PF	Nuc, Ag	L
**Sub-Sah. Afr.**	Benin (WHO)	Y	N	N	--	Y	N	Y	B, CSF	Nuc	PCR
	Cameroon (WHO)	Y	N	N	--	Y	N	Y	B, CSF	Nuc	PCR
	Ethiopia (WHO)	Y	N	N	--	Y	N	Y	B, CSF	Nuc	PCR
	Kenya, Asembo	Y	Y	Y	--	Y	Y	Y	B	--	Q, PCR
	Kenya, Kibera	Y	Y	Y	--	Y	Y	N	B	--	Q, PCR
	Kenya, Kilifi	Y	Y	Y	--	Y	Y	Y	B, CSF	--	Q, L, PCR
	Madagascar (WHO)	Y	N	N	--	Y	N	Y	B, CSF	Nuc	PCR
	Malawi, Blantyre District	Y	Y	Y	--	Y	Y	Y	B, CSF	--	L, PCR37
	South Africa	Y	Y	Y	Y	Y	Y	Y	B, CSF, PF	Nuc	Q, PCR38
	The Gambia, Basse	Y	Y	Y	Y	Y	Y	Y	B, CSF, PF	--	L, PCR21
	Zimbabwe (WHO)	Y	N	N	--	Y	N	Y	B, CSF	Nuc	PCR
**N. Afr. & W. Asia**	Israel	Y	Y	Y[7]	Y	Y	Y	Y	B, CSF	--	Q
	Morocco, Grand Casablanca	Y	Y	Y	--	Y	Y	N	B, CSF, PF	Nuc	Q, L, PCR70
**Asia**	Bangladesh	Y	N	Y[7]	Y[7]	Y[7]	Y	Y	B, CSF	Nuc, Ag	Q, PCR70
	Hong Kong	Y	Y	N	N	Y	Y	Y	B, CSF, PF	Nuc, Ag	PCR35
	Mongolia	Y	N	Y[7]	--	Y[7]	Y	Y	B, CSF, PF	Nuc, Ag	PCR70
	Japan	Y	Y	Y[7]	Y[7]	Y	Y	Y	B, CSF, PF	Nuc	Q, PCR70
	Singapore	Y	Y	Y	Y	Y	Y	Y	B, CSF, PF	--	Q
**Oceania**	Australia	Y	Y	Y	Y	Y	Y	Y	B, CSF	Nuc	Q, PCR
	Fiji	Y	Y	Y	--	Y	Y	Y	B, CSF, PF	Ag	Q, PCR70
	New Zealand	Y	Y	Y[7]	Y	Y	Y	Y	B, CSF, PF	Nuc, Ag	Q

[1] United Nations (UN) regions adapted from UN Statistics Division [5]. N. Am.: North America; LA & C: Latin America and the Caribbean; Sub-Sah. Afr.: Sub-Saharan Africa; N. Afr. & W. Asia: Northern Africa and Western Asia. [2] (WHO): WHO Global Invasive Bacterial Vaccine-Preventable Diseases (IB-VPD) Surveillance Network; (SIREVA): Pan American Health Organization Sistema de Redes de Vigilancia de los Agentes Responsables de Neumonias y Meningitis Bacterianas (SIREVA); (ECDC): The European Surveillance System (ECDC). [3] Availabilty of IPD data by age group and PCV period: -- PCV not universally used, no subsequent data; Y = Yes, data available; N = No, data not available. [4] Out of select specimen collection sites: B = Blood; CSF = cerebrospinal fluid; PF = pleural fluid. Some sites additionally indicated collection from "other" sterile sites that are not captured here. [5] Detection methods in addition to culture: -- indicates culture is the sole method of pneumococcal detection; Nuc = Nucleic acid detection; Ag = Antigen detection. [6] Where available, the number following "PCR" indicates the number of serotypes able to be identified by PCR: PCR37 = real-time polymerase chain reaction (21 assays/37 serotypes): serotypes detected vary by regional scheme (US [6], Latin America [7], Africa [8], and Asia [9]); PCR70 = conventional multiplex polymerase chain reaction (41 assays/70 serotypes): serotypes detected vary by regional scheme (US [10], Latin America [11], and Africa [12]); Q = Quellung reaction; L = Latex agglutination; WGS = Whole genome sequencing; NGS = Next generation sequencing; CST = Capsular sequence typing; GD = Gel diffusion. [7] Data only available for ages 0–17 years. [8] Clinical syndrome data are provided in SIREVA reports but are not serotype-specific, thus, not available in context relevant to table. [9] Incidence data are for pneumococcal meningitis only. [10] Adult IPD data available but not serotype-specific (serogrouped), therefore excluded from PSERENADE analyses. [11] Pneumococcal detection methods provided by ECDC reports [13–15]. Carriage studies suggest serotyping is determined through latex agglutination [16,17].

References

1. Pan American Health Organization; World Health Organization. Sistema Regional de Vacunas (SIREVA) II. Available online: https://www.paho.org/es/sireva (accessed on 31 August 2020).
2. Dirección Redes en Salud Pública; Subdirección Laboratorio Nacional De Referencia; Grupo de Microbiología. Vigilancia por Laboratorio de aislamientos invasores de Streptococcus pneumoniae Colombia 2006-2018: SIREVA II; Instituto Nacional de Salud: Bogotá, Colombia, 2019; pp. 1–16.
3. World Health Organization. Invasive Bacterial Vaccine Preventable Diseases Laboratory Network. Available online: http://www.who.int/immunization/monitoring_surveillance/burden/laboratory/IBVPD/en/ (accessed on 5 January 2021).
4. European Center for Disease Prevention and Control. The European Surveillance System (TESSy). Available online: https://www.ecdc.europa.eu/en/publications-data/european-surveillance-system-tessy (accessed on 7 January 2021).
5. United Nations. Methodology: Geographic Regions. Available online: https://unstats.un.org/unsd/methodology/m49/#geo-regions (accessed on 22 December 2020).
6. Centers for Disease Control and Prevention. *Protocol for the Triplex Real Time PCR - S. Pneumoniae Serotyping - Clinical Specimens - United States (US) Scheme*; 2014; pp. 1–4.
7. Centers for Disease Control and Prevention. *Protocol for the Triplex Real Time PCR - S. Pneumoniae Serotyping - Clinical Specimens - Latin America (LA) Scheme*; 2014; pp. 1–4.
8. Centers for Disease Control and Prevention. *Protocol for the Triplex Real Time PCR - S. Pneumoniae Serotyping - Clinical Specimens - Africa Scheme*; 2014; pp. 1–4.
9. Centers for Disease Control and Prevention. *Protocol for the Triplex Real Time PCR - S. Pneumoniae Serotyping - Clinical Specimens - Asia Scheme*; 2014; pp. 1–4.
10. Centers for Disease Control and Prevention. *Protocol for Multiplex PCR - S. Pneumoniae SEROTYPING - Clinical Specimens and Pneumococcal Isolates (USA Set)*; 2014; pp. 1–5.
11. Centers for Disease Control and Prevention. *Protocol for Multiplex PCR - S. Pneumoniae SEROTYPING - Clinical Specimens - Latin America Set*; 2014; pp. 1–5.
12. Centers for Disease Control and Prevention. *Protocol for Multiplex PCR - S. Pneumoniae SEROTYPING - Clinical Specimens and Pneumococcal Isolates - African Set*; 2014; pp. 1–5.
13. European Center for Disease Prevention and Control. *Invasive Pneumococcal Disease - Annual Epidemiological Report for 2018*; Stockholm: ECDC, 2020; p. 1.
14. European Center for Disease Prevention and Control. Surveillance Systems Overview for 2017. Available online: https://www.ecdc.europa.eu/en/publications-data/surveillance-systems-overview-2017 (accessed on 8 January 2021).
15. The European Commission. Commission Implementing Decision (EU) 2018/ 945 - of 22 June 2018 - on the Communicable Diseases and Related Special Health Issues to Be Covered by Epidemiological Surveillance as Well as Relevant Case Definitions. *Off. J. Eur. Union* **2018**, *1*, 74.
16. Usonis, V.; Stacevičienė, I.; Petraitienė, S.; Vaičiūnienė, D.; Alasevičius, T.; Kirslienė, J. Streptococcus Pneumoniae Nasopharyngeal Colonisation in Children Aged under Six Years with Acute Respiratory Tract Infection in Lithuania, February 2012 to March 2013. *Eurosurveillance* **2015**, *20*, 21079, doi:10.2807/1560-7917.ES2015.20.13.21079.
17. Stacevičienė, I.; Petraitienė, S.; Vaičiūnienė, D.; Alasevičius, T.; Kirslienė, J.; Usonis, V. Antibiotic Resistance of Streptococcus Pneumoniae, Isolated from Nasopharynx of Preschool Children with Acute Respiratory Tract Infection in Lithuania. *BMC Infect. Dis.* **2016**, *16*, 216, doi:10.1186/s12879-016-1544-9.
